# The use of parenteral application of ferric carboxymaltose in the prevention of iron deficiency anaemia in suckling piglets

**DOI:** 10.17221/22/2025-VETMED

**Published:** 2025-07-25

**Authors:** Martin Svoboda, Jan Vasek, Jiri Jarkovsky, Vojtech Skupien, Veronika Vitkova

**Affiliations:** ^1^Ruminant and Swine Clinic, Faculty of Veterinary Medicine, University of Veterinary Sciences Brno, Brno, Czech Republic; ^2^Institute of Biostatistics and Analyses, Faculty of Medicine, Masaryk University Brno, Brno, Czech Republic; ^3^Faculty of Veterinary Medicine, University of Veterinary Sciences Brno, Brno, Czech Republic

**Keywords:** carbohydrate complexes, haematocrit, haemoglobin concentration, swine

## Abstract

This study aimed to evaluate the effectiveness of ferric carboxymaltose injection in the prevention of anaemia in suckling piglets. In group A (*n* = 8), piglets were administered iron in the form of iron carboxymaltose complex (Ferinject^®^) at a dose of 200 mg i.m. (neck muscle) at the age of 3 days. In group B (*n* = 8), piglets were given iron in the form of iron dextran (Ferribion^®^) at a dose of 200 mg i.m. (neck muscle) at the age of 3 days. In group C (*n* = 8, anaemic control group), iron was not administered until the age of 10 days. During the study, no statistically significant differences were observed in the parameters of red blood cell count or blood plasma iron concentration between the group that received iron carboxymaltase and the group that received iron dextran. The results of haematological and biochemical examinations achieved in our study show that the application of iron carboxymaltase was successful in preventing iron deficiency anaemia in piglets. The results indicate comparable efficacy of iron carboxymaltase and iron dextran.

Iron deficiency anaemia is a serious problem in pig farming. The most common preventive measure is parenteral iron administration. Iron for parenteral administration is applied in the form of iron-carbohydrate complexes. Iron dextran is the most commonly used form of iron in suckling piglets. Traditionally, a dose of 200 mg Fe^3+^ i.m. is recommended for 2–3-day-old piglets (Maes et al. 2011). However, adverse effects have been reported following intramuscular administration of iron dextran in piglets (Svoboda et al. 2017). Iron dextran has also been used for parenteral iron administration in the treatment of anaemia in humans, and has been found to cause severe immunological reactions, including fatal anaphylactic reactions (Fishbane et al. 1996). For this reason, new safer alternatives have been developed. Ferric carboxymaltose (FCM) is a new form of iron for parenteral iron administration that does not contain dextran and is currently used in human medicine (Keating 2015).

However, there is no information on the efficacy and safety of FCM use in piglets in the available literature. The use of FCM in piglets could represent an alternative to the traditionally used iron dextran.

This study aimed to evaluate the effectiveness of ferric carboxymaltose injection in the prevention of anaemia in suckling piglets.

## MATERIAL AND METHODS

The experiment took place at the university farm in Nový Jičín in the Czech Republic. A total of 24 piglets (Czech Large White and Landrace) from three litters were used. Each piglet was marked with one coloured ear tag in the right earlobe before the start of the experiment. The experiment used so-called split litters, i.e., the piglets in each litter were randomly divided (randomisation) into three groups.

In group A (*n* = 8), piglets were administered iron in the form of iron carboxymaltose complex (Ferinject^®^) at a dose of 200 mg i.m. (neck muscle) at the age of 3 days. In group B (*n* = 8), piglets were given iron in the form of iron dextran (Ferribion^®^) at a dose of 200 mg i.m. (neck muscle) at the age of 3 days. In group C (*n* = 8, anaemic control group), iron was not administered until the age of 10 days. At the age of 10 days, these piglets were given iron therapeutically in the form of iron dextran at a dose of 200 mg i.m. (gluteal muscle). After the administration of iron, the piglets in all groups were clinically monitored for the occurrence of adverse effects. The piglets were weaned at the age of 28 days. Piglets were offered weaning pellets with an iron content of 200 mg.kg^–1^ from 7 days of age (Pig Starter super gr.; MIKROP ČEBÍN a.s., Čebín, Czech Republic).

Samples for haematological and biochemical examination were collected at the age of 3, 10, and 24 days from the *vena cava cranialis*. Piglets were weighed each time during blood sampling. EDTA was used as an anticoagulant when taking samples for haematological examination. Heparin was an anticoagulant when taking a sample for biochemical examination.

### Haematological analysis

The analysis included the red blood cells (RBC), haemoglobin concentration (HGB), packed cell volume (PCV), mean corpuscular volume (MCV), mean corpuscular haemoglobin (MCH), and mean corpuscular haemoglobin concentration (MCHC).

The Mindway, BC–2800 Vet. device (Guangzhou Medsinglong Medical Equipment Co., Ltd., Guangdong, P.R. China) was used for the haematological analysis. The Dia Rinse D, Dia Lyse Diff D-CF, Dia EZ Cleanser D, and Probe Cleanser (Medesa, Polička, Czech Republic) were used as flush solutions.

The colorimetric method was used for the detection of HGB. The impedance was used for other variables in our study.

### Biochemical analyses

The biochemical examination included determination of iron concentration in blood plasma. The method of atomic absorption spectrometry was used. The Solaar (Thermo Electron Corporation, Waltham, MA, USA) machine was used for the determination of iron in plasma with a limit of quantification (LOQ) of 1 μmol.l^–1^. For statistical calculations, the values below the LOQ were replaced by half of this limit, i.e., by the value of 0.05 μmol.l^–1^.

### Statistical analysis

The results are expressed as mean and standard deviation. The Kruskal-Wallis test was used to test the statistical significance of the differences among the groups. It was followed by the Mann-Whitney test for between-group comparison (*P* < 0.05). Friedman’s test, followed by Wilcoxon’s test, was used to analyse the statistical significance of the differences among and between the time points (*P* < 0.05). The analyses were done using SPSS v28.0.1.1 (IBM, Armonk, NY, USA).

## RESULTS

### Comparison of haematological and biochemical variables among groups

The results are presented in Table 1. During the study, no statistically significant differences were noted in the red blood cell indicators between the group that received iron carboxymaltose and the group that received iron dextran. As expected, the values of red blood cell indicators in the anaemic control group were significantly lower than in the groups in which iron was administered at the age of 3 days.

**Table 1:** Results of body weights, plasma iron concentrations and red blood cell indicators

Indicators	Day 3				Day 10				Day 24		
					groups						
	A (*N* = 8)	B (*N* = 8)	C (*N* = 8)		A (*N* = 8)	B (*N* = 8)	C (*N* = 8)		A (*N* = 8)	B (*N* = 8)	C (*N* = 8)
Weight (kg)	1.9 ± 1.2^a^	1.8 ± 1.8^a^	1.8 ± 2.8^a^		4.0 ± 1.9^a^	3.8 ± 2.1^a^	3.5 ± 1.1^a^		10.2 ± 1.7^a^	8.8 ± 1.5^a^	10.1 ± 3.0^a^
Iron (μmol/l)	2.2 ± 1.0^a^	1.8 ± 0.5^b^	1.0 ± 0.6^b^		22.5 ± 0.5^a^	17.6 ± 0.6^a^	0.5 ± 0.5^b^		6.5 ± 0.4^a^	6.6 ± 0.5^a^	6.5 ± 0.4^a^
Erythrocytes (× 10^12^/l)	4.5 ± 6.7^a^	4.7 ± 3.2^a^	4.5 ± 3.7^a^		5.4 ± 1.7^a^	5.3 ± 2.3^a^	3.9 ± 1.3^b^		29.0 ± 2.6^a^	29.2 ± 2.4^a^	27.6 ± 1.5^a^
Haemoglobin (g/l)	82.3 ± 3.2^a^	87.6 ± 2.6^a^	84.6 ± 3.4^a^		105.9 ± 5.3^a^	103.5 ± 5.4^a^	55.4 ± 4.4^b^		44.6 ± 3.4^a^	44.6 ± 3.5^a^	43.0 ± 3.2^b^
Haematocrit (%)	33.0 ± 0.8^a^	35.1 ± 1.1^a^	33.8 ± 0.8^a^		31.7 ± 1.4^a^	31.4 ± 1.9^a^	16.1 ± 2.1^b^		15.9 ± 1.5^a^	15.5 ± 1.4^a^	14.5 ± 1.2^a^
Mean corpuscular volume (MCV) (fl)	73.5 ± 9.6^a^	74.4 ± 12.3^a^	75.5 ± 11.3^a^		59.7 ± 11.6^a^	59.6 ± 15.9^a^	41.9 ± 25.4^b^		356.1 ± 13.6^a^	347.8 ± 13.7^a^	338.8 ± 5.2^a^
Mean corpuscular haemoglobin (MCH) (pg)	18.3 ± 79.2^a^	18.6 ± 144.0^a^	18.8 ± 151.4^a^		19.8 ± 113.8^a^	19.6 ± 157.0^a^	14.4 ± 628.0^b^		548.5 ± 71.8^a^	524.1 ± 91.0^a^	598.4 ± 170.1^a^
Mean corpuscular haemoglobin concentra-tion (MCHC) (g/l)	248.9 ± 5.2^a^	249.4 ± 2.8^a^	249.9 ± 3.1^a^		334.1 ± 6.5^a^	329.5 ± 2.2^a^	343.4 ± 1.3^a^		23.1 ± 3.3^a^	22.5 ± 2.3^a^	22.7 ± 1.9^a^

^a,b^Means with the same blood collection time and row lacking a common superscript letter differ significantly (*P* < 0.05)

The results are presented as the mean and a standard deviation

Group A = iron carboxymaltose complex; group B = iron dextran; group C = control

At the age of 10 and 24 days, no piglets with haemoglobin values lower than 90 g^–1^ per litre were found in the iron carboxymaltose group.

In the group that received iron dextran, only one piglet was found with a value lower than 90 g.l^–1^ (88 g.l^–1^) on the 24^th^ day of life.

Likewise, the iron concentrations in the blood plasma did not differ significantly between the groups that received iron carboxymaltose and iron dextran throughout the experiment. The iron concentrations in the blood plasma in the anaemic control group were significantly lower than in the groups in which iron was administered at the age of 3 days.

### Development of haematological and biochemical indicators within the individual groups over time

The results are presented in Table 1. Seven days after iron administration, which took place on day 3 of age, there was a significant increase in haemoglobin concentration, haematocrit, red blood cell count, mean corpuscular haemoglobin concentration (MCHC), and plasma iron concentration in the iron carboxymaltose and iron dextran-treated groups. A decrease in mean corpuscular volume (MCV) was noted in both groups on day 10 of age. The erythrocyte haemoglobin (MCH) increased significantly in the carboxymaltose group.

In the anaemic control group, there was a decrease in haemoglobin concentration, haematocrit, red blood cell count, erythrocyte haemoglobin (MCH), mean corpuscular volume (MCV), and iron concentration between days 3 and 10 of age. In the anaemic control group, the mean corpuscular haemoglobin concentration (MCHC) increased on day 10 of age. In this group, the application of iron in the form of dextran on the 10^th^ day of age increased haemoglobin concentration, haematocrit, red blood cell count, erythrocyte haemoglobin (MCH), mean corpuscular volume (MCV), and iron concentration on the 24^th^ day of age.

Both groups in which iron was administered on day 3 (iron carboxymaltose, iron dextran) experienced a significant decrease in blood plasma iron concentration between the 10^th^ and 24^th^ day.

### Clinical observations

No adverse effects were observed after iron administration.

### Comparison of body weights

The results are presented in Table 1. No statistically significant differences in achieved body weights were found between the individual groups during the experiment.

## DISCUSSION

Iron for parenteral administration is applied in the form of iron-carbohydrate complexes. They consist of a mineral core surrounded by a carbohydrate shell.

The function of the carbohydrate shell is to stabilise the complex and reduce the risk of free iron release, which can be the cause of adverse effects (Keating 2015). Free iron ions catalyse the formation of free oxygen radicals. This induces lipoperoxidation in cell membranes (Hirayama and Yasutake 1998).

Upon administration, the complexes are taken up by reticuloendothelial macrophages. Different forms of iron for parenteral application differ in the stability of the complex, their effectiveness, and the occurrence of adverse effects (Evstatiev et al. 2011; Geisser and Burckhardt 2011).

Several adverse effects have been described with the use of iron dextran in pigs. Patterson et al. (1971) demonstrated an increase in lipid peroxidation levels in the gluteal muscles of piglets that died after iron dextran injection.

Lipoperoxidation also increases the organism’s susceptibility to infections (Morris et al. 1995). For example, a higher incidence of polyarthritis in piglets treated with injectable iron dextran was demonstrated (Holmgren 1996).

With a significant lack of antioxidants (selenium, vitamin E), cases of fatal intoxication have also been described in piglets after iron detran injection (Suveges and Glavits 1976; Kolb and Hoffmann 1989).

Thanks to its structure, FCM is more stable than previously used preparations. It is composed of a core of ferric hydroxide stabilised by a carbohydrate coating. This form enables the controlled delivery of iron to the cells of the reticuloendothelial system (Koduru and Abraham 2016).

In human patients, FCM was found to show higher efficacy and safety compared to previously used preparations (iron sucrose) (Evstatiev et al. 2011). A study comparing the efficacy and safety of FCM with iron dextran in human patients showed comparable efficacy between the two groups, with fewer adverse effects in the FCM group (Hussain et al. 2013).

Different authors use different haemoglobin concentrations to evaluate anaemia. Egeli et al. (1998) state that the anaemic limit, i.e., the point when anaemia begins to exert a detrimental effect on weight gain or gives rise to clinical symptoms of anaemia, is at a haemoglobin concentration of 80 g.l^–1^. Bhattarai and Nielsen (2015) are stricter; they consider a haemoglobin concentration of below 90 g.l^–1^ as indicative of anaemia. In our study, we decided to divide the piglets into anaemic and non-anaemic according to the haemoglobin threshold of 90 g.l^–1^.

The results of haematological examination achieved in our study show that the application of iron carboxymaltose was successful in preventing iron deficiency anaemia in piglets. The results indicate comparable efficacy of iron carboxymaltose and iron dextran.

However, there was a significant drop in the blood plasma iron concentrations between days 10 and 24, and the measured values reached very low levels. Kaneko (1993) provided reference values of 21.7 ± 5.9 μmol.l^–1^ for serum iron concentrations in piglets.

Our results of the blood plasma iron concentrations indicate a latent iron deficiency and the need for repeated iron administration. A repeated dose of iron is recommended, for example, by Haugegaard et al. (2008). According to their results, a second injection at the age of 21 days improved red blood cell indices and increased weight gain by 25 g.

Currently, the limitation for the use of this preparation is its high price and the fact that this product is not yet registered for use in pigs.
